# Approaches for Modeling of Intensive Longitudinal Data to Understand Cognitive Aging

**DOI:** 10.1093/geroni/igaa057.1867

**Published:** 2020-12-16

**Authors:** Karra Harrington, Nelson Roque, Jacqueline Mogle

## Abstract

Understanding age-related change in cognition and identification of pathological changes requires sensitive and valid measurement of cognitive performance across time. Technological advances, such as ambulatory assessment of cognition using smartphones, have enabled intensive longitudinal methods where data is collected with many measurements over time. Our research group has developed novel ambulatory assessments that provide reliable, sensitive, and ecologically valid measurement of cognition across multiple timescales; from momentary changes to change across years. This symposium will present a spectrum of approaches to analysis of intensive longitudinal data that can inform models of cognitive aging. All three presentations will draw on data from measurement burst studies that apply our ambulatory cognitive assessment methods in community-based samples (i.e., systematically recruited in the Bronx, New York). For each measurement burst, participants undergo assessment consisting of brief surveys and cognitive tests via smartphone, up to 7 times per day across 14 days. Oravecz et al. will discuss the application of a Bayesian multilevel implementation of the double exponential model to account for retest effects while quantifying change in peak cognitive performance across time. Kang et al., will demonstrate a growth curve modeling approach for assessing the effects of between-person variables (i.e., loneliness) on change in cognition across measurement bursts. Harrington et al., will demonstrate a model-based cluster analysis approach, leveraging ambulatory assessments of subjective and objective cognitive function to unpack latent groups as a function of age and loneliness. Measurement, Statistics, and Research Design Interest Group Sponsored Symposium.

